# 

**Published:** 1908-06-20

**Authors:** 


CONTENTS.

PAGE

**The Medical Examination of Employees** ... ... 299
**The Faculty of the Investigator** ... ... ... ... 300
**Annotations—**
The Proposed Bombay Medical Congress—"Crowner's Law"—
The Opponents of our Profession ... ... ... ... ... 301
**Medical Opinion and Movement** ... ... ... ... 302
**Hospital Clinics—**
On Diseases of the Pancreas. By Dr. Albu ... ... ... 305
**Special Article—**
The Treatment of Acute Intussusception ... ... ... ... 306
**Medicine—**
Dietl's Crises ... ... ... ... ... ... ... ... ... 309
The Sequelæ of Enteric Fever—III. ... ... ... ... ... 310
**Points in Surgery—**
Some Aspects of Intestinal Obstruction ... ... ... ... 311
Coxa Vara ... ... ... ... ... ... ... ... ... ... 312

PAGE

**Laryngology and Rhinology—**

Nasal Obstruction—I. ... ... ... ... ... ... ... ... 313

**Medico-Legal Points—**

Abortion from a Legal Point of View... ... ... ... ... 314

**Anæsthetics—**

Massage of the Heart ... ... ... ... ... ... ... ... 315

**Diseases of Children—**

The Refusal of Food in Infancy ... ... ... ... ... ... 316

**"The Hospital" Medical Book Supplement.—No. VIII.** ... ... ... ... ... ... 317

**Hospital Administration—**

Sheffield's Opportunity ... ... ... ... ... ... ... 321

News and Coming Events ... ... ... ... ... ... ... 323

**New Appliances and Things Medical** ... ... ... ... 324

**Nursing Administration—**

Recommendations and Testimonials... ... ... ... ... 325

**The Graduates' Medical Schools** ... ... ... ... 326

